# Prevalence and influencing factors of post-traumatic stress disorder among Chinese healthcare workers during the COVID-19 epidemic: a systematic review and meta-analysis

**DOI:** 10.3389/fpsyt.2024.1323111

**Published:** 2024-02-15

**Authors:** Min Zhang, Mingyu Bo, Huixin Wang, Wenyi Fan, Lingling Kong, Chunjie Zhou, Zhenxing Zhang

**Affiliations:** ^1^ Department of Applied Psychology, Binzhou Medical University, Yantai, Shandong, China; ^2^ School of Information and Electrical Engineering, Ludong University, Yantai, Shandong, China

**Keywords:** post-traumatic stress disorder, COVID-19, healthcare workers, influencing factors, meta-analysis

## Abstract

**Background:**

Post-traumatic stress disorder is an important psychological problem affecting the physical mental health of Chinese healthcare workers during the COVID-19 pandemic.

**Aims:**

To estimate the prevalence and influencing factors of post-traumatic stress disorder (PTSD) among Chinese healthcare workers during COVID-19.

**Methods:**

Search of Chinese and English literature in PubMed, EMbase, Web of Science, Medline, Elsevier, SpringerLink, China Biomedical Literature Database, CNKI, Wan-fang, and CQVIP for the period from December 2019 to August 2023. Stata 14.0 software was used for data analysis. The methodological quality of each study was scored, and data were extracted from the published reports. Pooled prevalence was estimated using the Random-effects model. Publication bias was evaluated using Egger’s test and Begg’s test.

**Results:**

Twenty-one studies included 11841 Chinese healthcare workers in this review. First, the overall prevalence of Post-traumatic stress disorder among Chinese healthcare workers during the COVID-19 epidemic was 29.2% (95% CI: 20.7% to 33.7%). Twelve factors included in the meta-analysis were found to be protective against PTSD among Chinese healthcare workers: female, nurse, married, front-line work, less work experience, family or friend diagnosed with COVID-19, history of chronic disease and fear of COVID-19. Conversely, outside Hubei, higher education, social support and psychological resilience are protective factors.

**Conclusion:**

These recent findings increase our understanding of the psychological status of Chinese healthcare workers and encourage that long-term monitoring and long-term interventions should be implemented to improve the mental health of Chinese healthcare workers in the aftermath of the COVID-19.

## Introduction

Beginning in late 2019, a novel infectious disease caused by Severe Acute Respiratory Syndrome Coronavirus 2 (SSARS-CoV-2) first broke out in Wuhan, China (1). In March 2020, the World Health Organization officially named this virus-induced disease Corona Virus Disease 2019 (COVID-19), characterized by rapid transmission, widespread, and high contagiousness (2). As of April 10, 2023, more than 762 million cases of SARS-CoV-2 coronavirus have been reported, with 6.89 million deaths (3). The rapid spread of this infection, with its high exposure and mortality rates, has impacted China’s healthcare system, creating a long-term mental health burden for the public and increasing the demand for Chinese healthcare workers (HCWs) (4).

Healthcare workers, as the frontline force in epidemic prevention and control, face a greater risk of infection than other occupational groups because of overwork caused by emergencies, which has caused Chinese healthcare workers to face unprecedented challenges and pressures, and some of them have even developed symptoms of post-traumatic stress disorder (PTSD) (5). According to the Diagnostic and Statistical Manual of Mental Disorders, Version V (DSM-5), the diagnosis of PTSD requires exposure to traumatic events followed by persistent symptoms and injuries that impair their social functioning. According to DSM-5, in order to meet the diagnostic criteria for PTSD, one must directly experience (standard A1) or witness (standard A2) traumatic events, including actual or threatening death, serious injury, or sexual violence, except for indirect contact (e. g., sudden violent or accidental death of a loved one; standard A3) or traumatic experience (e. g., first responder collecting remains; standard A4) (6).

Since 2020 and until April 2023, China has been adopting a strict epidemic prevention and control policy and continuously strengthening its healthcare service system (7), during which healthcare workers have been subjected to tremendous work pressure and psychological stress, making them more prone to symptoms of post-traumatic stress disorder (PTSD). Previous evidence on coronavirus epidemics and preliminary findings from the COVID-19 pandemic have highlighted its psychological impact on healthcare workers (8–10). Some studies have also shown that healthcare workers in China are more likely to develop PTSD symptoms than the general population (11, 12). Therefore, there is an urgent need to prevent and treat PTSD among Chinese healthcare workers.

China was the first country to discover the novel coronavirus pneumonia epidemic and has an active psychosocial academic community that has conducted a great deal of research on it. Currently, there have been worldwide systematic reviews and meta-analysis of depression, anxiety, post-traumatic stress disorder and other mental health disorders during the COVID-19 pandemic (13–15). However, related studies focused before 2021, lack of search of relevant Chinese databases and no comprehensive analysis of Chinese medical personnel. In addition, it is also influenced by confounding factors such as sample size, so the accuracy of the study results is questionable. Currently, we need reliable and comprehensive estimates of psychological symptoms among Chinese healthcare workers during COVID-19 to inform their preventive and treatment actions. Meanwhile, in order to explore the prevalence of PTSD among Chinese healthcare workers during COVID-19 and to improve the prognosis, it is necessary to understand the influencing factors, with a view to providing a guiding basis for alleviating the clinical symptoms of PTSD.

## Methods

### Search strategy

This meta-analysis is reported following the Preferred Reporting Items for Systematic Reviews and Meta-analyses (PRISMA) checklist and was registered in the PROSPERO database (International Prospective Register of Systematic Reviews).In this study, we systematically searched Chinese and English databases such as PubMed, EMbase, Web of Science, Medline, Elsevier, SpringerLink, China Biomedical Literature Database, China Biomedical Literature Database, CNKI, Wan-fang, and CQVIP to screen cross-sectional studies on the influencing factors of post-traumatic stress disorder (PTSD) in Chinese healthcare workers during the COVID-19 period, which lasted from December 2019 to August 2023, with a combination of subject and free-form words as the search strategy. For MEDLINE, the terms are: (“COVID-19” or “2019 novel coronavirus” or “novel coronavirus pneumonia”) and (“Stress Disorders” or “Post-Traumatic” or “Post- traumatic stress disorder” or “PTSD” or “Stress Disorders”) and (“Healthcare workers” or “Physicians” or “Nurses” or “Medical personnel” or “Emergency Department Personnel”) and (“Influencing factors” or “Protective factors” or “Factors”). The search strategy was discussed and decided upon by the three investigators.

### Inclusion and exclusion criteria

Inclusion and exclusion criteria were predetermined prior to literature screening. Inclusion criteria (1): Chinese and English studies since the outbreak of COVID-19 in December 2019 (2); the type of study was cross-sectional; (3) the measurement tools used were specific and clear; (4) only one of the duplicated publications was taken;(5)Chinese and English literature for studies of Chinese medical workers. Exclusion Criteria: (1) Literature in languages other than English and Chinese; (2) Literature with missing data; (3) Literature of poor quality in English and Chinese; (4) Literature in the category of reviews, conferences and guidelines.

### Study selection and data extraction

Two researchers alone extracted information, screened the literature and cross-checked, with a third researcher assisting in case of disagreement Judgment. All databases were fully searched and the required literature was screened using EndNote version 9.3 software. Inclusion was determined by further reading of abstracts and full texts after excluding duplicates and obviously irrelevant literature. Data extraction included the following information: (i) First author, (ii) year of publication, (iii) subjects, (iv) sample size, (v) prevalence of PTSD, (vi) PTSD assessment tool, (vii) influencing factors, (viii) evaluation of literature quality.

### Assessment of quality

Assessed using the Agency for Healthcare Research and Quality (AHRQ) cross-sectional study evaluation criteria (16), A total of 11 entries were included, with a total score of 11 points, 3 evaluation options for each entry, 1 point for “yes”, 0 points for “unclear” or “no”, and ≤3 points for low-quality literature. In this study, if the total score was ≥6, the literature could be included in the Meta-analysis.

### Statistical analysis

The meta-analysis was performed using the Stata 14.0 software. First, heterogeneity was analyzed using the Q-test and the I² statistic, and OR and 95% CI were used as effect indicators; if P > 0.1 and I²≤50%, it means that the heterogeneity is small and a fixed-effects model is used; conversely, if the heterogeneity is large, a random-effects model is used. In addition, a sensitivity analysis was performed to assess whether the exclusion of any of the studies included produced significant changes in outcome. The publication bias was evaluated by Begg’s and Egger’s tests on funnel plots. In the analysis, differences were considered statistically significant at P<0.05.

## Results

### Study selection

**Figure 1:**
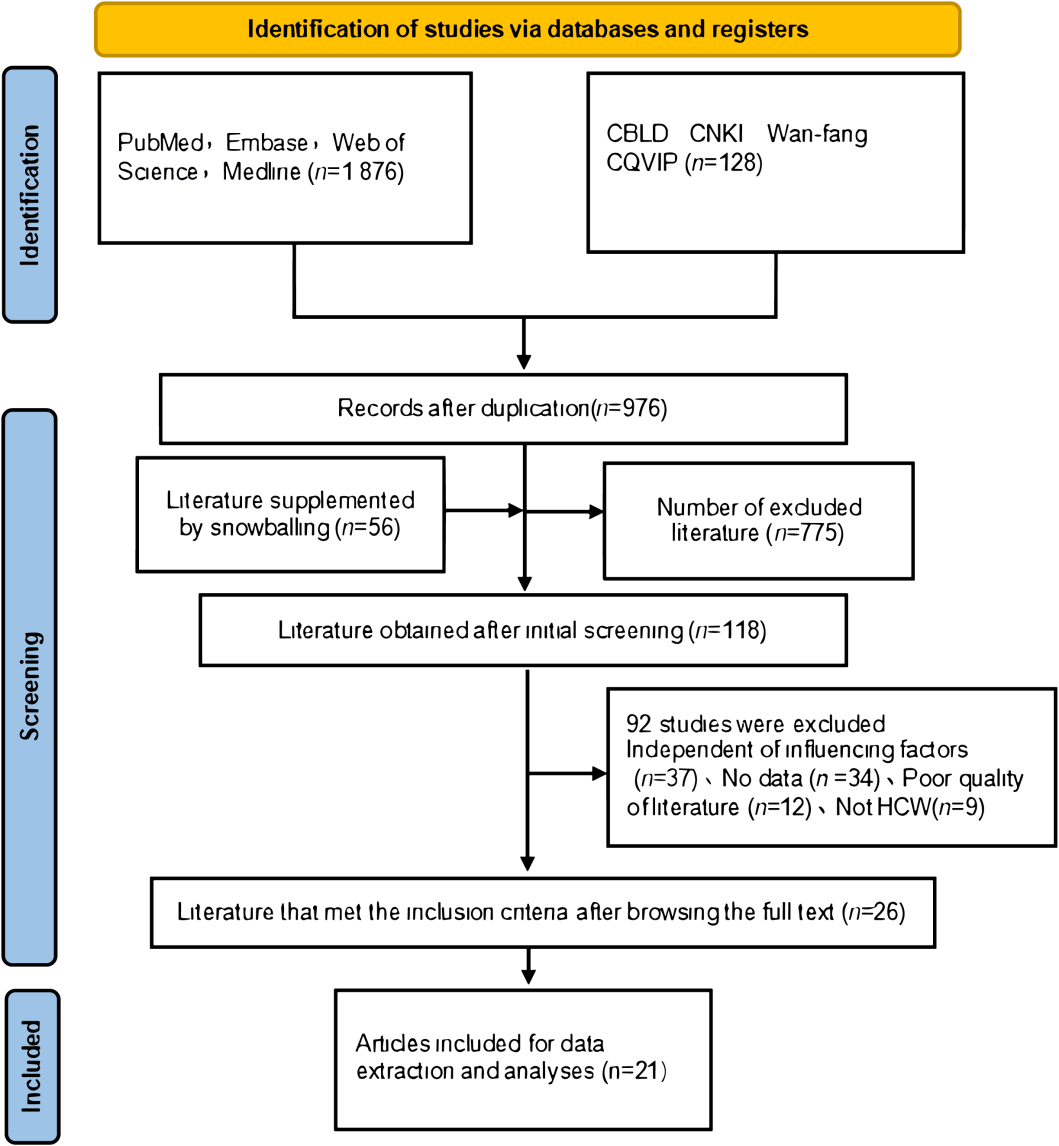
Research screening flow chart.

During the initial database search, 2004 records were identified as being related, and 976 articles were left after deleting duplicates. The titles and abstracts were initially screened to remove articles that were not relevant to the content of the review articles. Finally, 21 studies were included in the systematic review and meta-analysis (17–37). Of the 21 studies included in this analysis, six of them were published in Chinese journals by Chinese authors, and the other 15 studies were published by Chinese authors and by foreign universities, all included data related to Chinese medical staff. As shown in **Figure 1**, the selection process for the study is summarized as follows.

### Study characteristics

The main characteristics of the 21 included studies are shown in **Table 1**. Twenty-one cross-sectional studies published in 2019 – 2023 reported the prevalence and associated factors of PTSD among Chinese healthcare workers during COVID-19, involving 11841 study participants and 17 influencing factors, which were extracted if ≥3 papers mentioned the same influencing factor. A total of 12 influential factors were extracted in this study: Female, nurse, married, front-line work, fewer years of working experience, family or friend diagnosed with COVID-19, history of chronic disease, fear of COVID-19, high education, outside of Hubei province, social support, psychological resilience. There were 5 Chinese-language and 16 English-language papers in this study. Most of the literature used the revised version of the Impact of Events Scale (IES-R) as a diagnostic assessment tool (42.86%). In contrast, the rest of the literature used the Posttraumatic Stress Disorder Checklist-City Resident Version (PCL-C), and the Posttraumatic Stress Disorder Checklist for DSM-5 (PCL-5) to diagnose PTSD in Chinese healthcare workers during the COVID-19 pandemic. The quality of the literature was moderate to high.

**Table 1:** Characteristics of included studies.

First author	Year	Survey object	Sample size	Disease rate	Survey tools	Influencing factors	Quality score
Tang (17)	2023	HCWs	3762	53.80%	PCL-C	5	8
Niu (18)	2022	Frontline HCWs	187	11.20%	PCL-C	1, 8	7
Zhang (19)	2020	Nurses	717	57.70%	IES-R	5, 11	6
Zhe (20)	2022	HCWs	8316	23.30%	IES-R	2, 7	8
Cheng (21)	2022	HCWs	2192	75.55%	IES-R	6, 9	7
Song (22)	2020	Frontline Nurses	14825	9.10%	PCL-5	2, 5, 7, 10	8
Yang (23)	2022	HCWs	1993	9.30%	PCL-5	2	7
Zhang (24)	2020	HCWs	642	20.87%	PCL-C	11	6
Zhang (25)	2021	HCWs	421	13.20%	IES-R	3	7
Nie (26)	2020	Frontline Nurses	263	25.10%	IES-R	5	6
Li (27)	2020	Frontline Nurses	356	61.80%	PCL-5	3, 7, 12	7
Yin (28)	2020	HCWs	377	3.80%	PCL-5	1, 7	6
Guo (29)	2021	Frontline HCWs	1091	11.00%	PCL-C	10	7
Li (30)	2023	HCWs	425	43.29%	IES-R	4, 6, 7, 10	7
Zhou (31)	2022	Frontline Nurses	757	13.50%	PCL-5	6, 12	7
Wang (32)	2021	HCWs	1897	9.80%	IES-R	2, 4, 8	8
Jing (33)	2022	HCWs	443	14.40%	PCL-C	7, 8, 12	6
Pan (34)	2021	HCWs	659	13.70%	PCL-5	9, 11	6
Xia (35)	2021	Nurses	1728	39.12%	PCL-5	3, 8	7
Lai (36)	2020	HCWs	1257	71.50%	IES-R	1, 4, 10	7
Li (37)	2020	HCWs	4369	31.60%	IES-R	2, 5, 6, 9	8

Influencing factors: 1=Female, 2=Nurse, 3=Married, 4=First line of work, 5=Fewer years of working experience, 6=Family member or friend with a diagnosis of COVID-19, 7=History of chronic illness, 8=Fear of COVID -19 fear, 9=highly educated, 10=outside Hubei province, 11=social support, 12=psychological resilience.

IES-R, Incident Effects Scale Revised; PCL-C, Posttraumatic Stress Disorder Checklist-City Resident Version; PCL-5, DSM-5’s Posttraumatic Stress Disorder Checklist.

### Risk of bias in studies

Two independent reviewers assessed the included studies using the Agency for Healthcare Research and Quality (AHRQ) cross-sectional study evaluation criteria. All articles met quality requirements and were included in this systematic review and meta-analysis.

### Meta-analysis results

**Figure 2:**
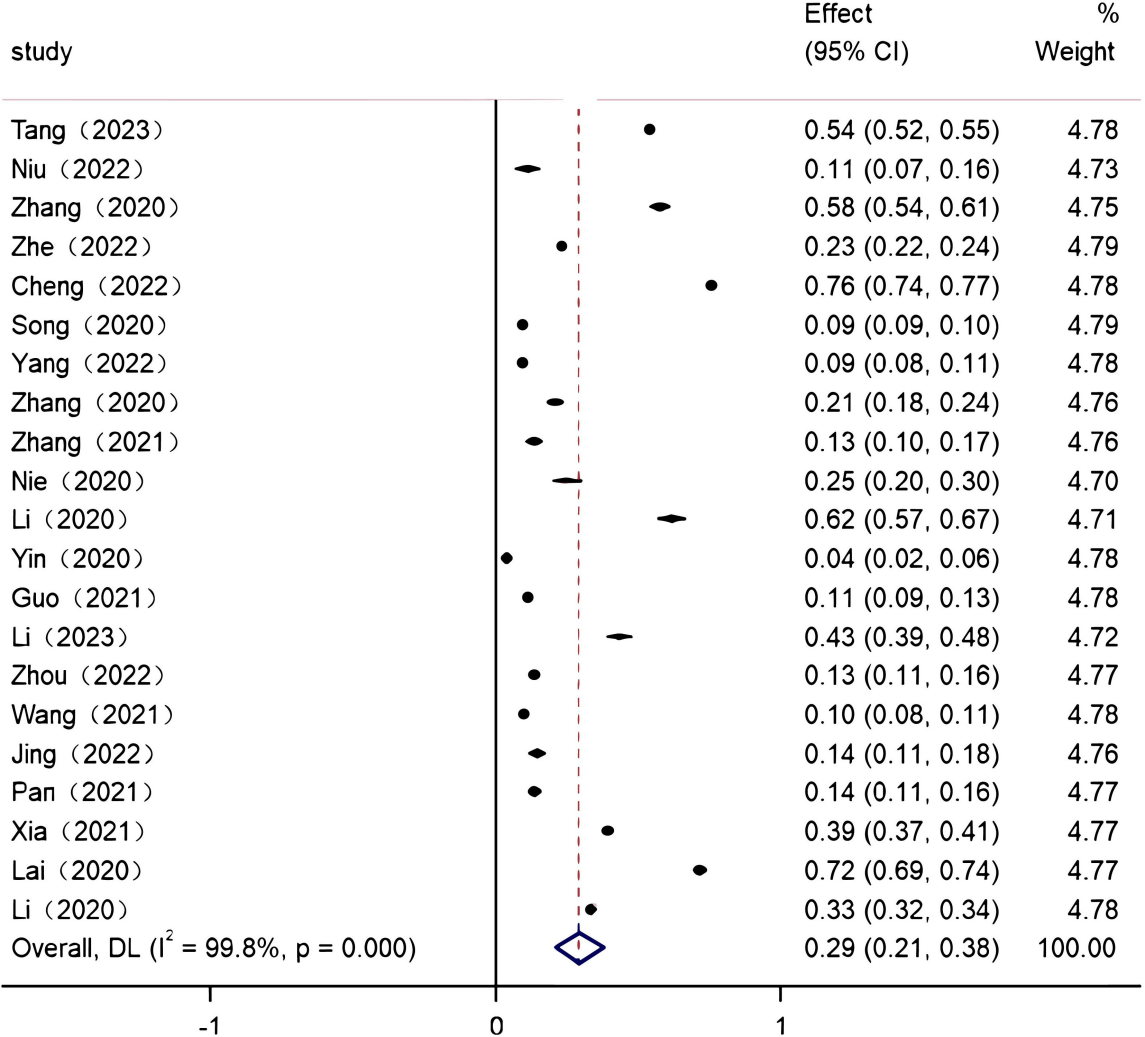
Forest plot of the prevalence of post-traumatic stress disorder among Chinese healthcare workers during the COVID-19 pandemic.

Due to the obvious heterogeneity among the 21 individual studies included, a random-effects model was chosen to combine the effect values. The results showed that the overall prevalence of PTSD among Chinese healthcare workers during the COVID-19 pandemic was 29.2% (95% CI: 20.7%-37.7%), The analysis results are shown in forest plots in **Figure 2**.

Meta-analysis was performed on 12 influencing factors, of which 8 influencing factors, namely female, married, fewer years of working experience, fear of COVID-19, high education, outside of Hubei province, social support, and psychological resilience, were heterogeneous among the studies (I²>50%, P<0.10), and therefore Meta-analysis was performed using a random-effects model; of which 4 influencing factors, namely nurses, first-line work, family members or friends with a confirmed diagnosis of COVID-19, and history of chronic diseases, the four influencing factors were not heterogeneous among the studies (I²≤50%, P≥0.10), so Meta-analysis was performed using a fixed-effects model. The results showed that high education, outside Hubei province, social support, and psychological resilience were protective factors for PTSD among Chinese healthcare workers during COVID-19 (P<0.01). And female, nurse, married, frontline work, fewer years of working experience, family or friend diagnosed with COVID-19, history of chronic disease, and fear of COVID-19 were Chinese healthcare workers’ risk factors for PTSD (P ≤ 0.01, **Table 2**).

**Table 2:** Meta-analysis of factors influencing PTSD among Chinese healthcare workers during the COVID-19 pandemic.

Influencing factors	Number of publications	Heterogeneity test		Modelling	Combined effect size	
		*P*	*I²*(%)		*OR*(95%CI)	*P*
Female	3	0.058	64.80	Random effects model	2.02(1.27~3.20)	<0.01
Nurses	5	0.335	12.30	Fixed effects model	1.58(1.44~1.73)	<0.01
Married	3	<0.01	79.4	Random effects model	4.48(2.00~10.02)	<0.01
Front-line work	3	0.242	29.5	Fixed effects model	1.83(1.54~2.19)	<0.01
Fewer years of working experience	5	<0.01	79.2	Random effects model	1.32(1.08~1.62)	<0.01
Family or friend diagnosed with COVID-19	3	0.192	39.4	Fixed effects model	1.89(1.33~2.70)	<0.01
History of chronic illness	3	0.612	0	Fixed effects model	1.62(1.40~1.87)	<0.01
Fear of COVID-19	4	<0.01	87.4	Random effects model	1.81(1.42~2.89)	0.012
High education	4	0.015	71.5	Random effects model	0.58(0.37~0.91)	<0.01
Outside Hubei province	4	0.086	54.5	Random effects model	0.54(0.41~0.72)	<0.01
Social support	3	<0.01	94.6	Random effects model	0.44(0.17~1.15)	<0.01
Psychological resilience	3	<0.01	80.4	Random effects model	0.97(0.91~1.02)	<0.01

### Sensitivity analysis

Sensitivity analysis of the 12 influencing factors was performed using fixed- and random-effects models(combined effect test, P < 0.05).The results showed that the random effects and fixed effects model analyses of the 12 influencing factors were close to each other, indicating that the results of this study are robust (**Table 3**). Eight of the influencing factors showed heterogeneity, and the combined results did not change in direction after excluding one class of literature. This finding indicated good stability of the pooled results and high reliability of the conclusion (**Table 4**).

**Table 3:** Sensitivity analysis of factors influencing PTSD among Chinese healthcare workers during the COVID-19 pandemic[OR (95% CI)].

Influencing factors	Random effects model	Fixed effects model
Female	2.02(1.27~3.20)	1.76(1.39~2.22)
Nurses	1.57(1.41~1.75)	1.58(1.44~1.73)
Married	4.48(2.00~10.02)	4.68(3.25~6.74)
Front-line work	1.86(1.50~2.31)	1.83(1.54~2.19)
Fewer years of working experience	1.32(1.08~1.62)	1.07(1.03~1.12)
Family or friend diagnosed with COVID-19	1.89(1.33~2.70)	1.85(1.41~2.44)
History of chronic illness	1.62(1.40~1.87)	1.62(1.40~1.87)
Fear of COVID-19	1.81(1.42~2.89)	1.22(1.13~1.31)
High education	0.58(0.37~0.91)	0.78(0.68~0.89)
Outside Hubei province	0.54(0.41~0.72)	0.57(0.47~0.69)
Social support	0.44(0.17~1.15)	0.93(0.90~0.96)
Psychological resilience	0.97(0.91~1.02)	0.97(0.96~0.99)

**Table 4:** Exclusion analysis of factors affecting PTSD among Chinese healthcare workers during the COVID-19 pandemic.

Influencing factors	Exclusion of literature	Preclusion			After elimination		
		Modelling	*OR*(95%*CI*)	P	Modelling	*OR*(95%*CI*)	*P*
Female	(Li, 2020)	Random effects model	2.02(1.27~3.20)	<0.01	Fixed effects model	2.41(1.64~3.54)	<0.01
Married	(Yang, 2022)	Random effects model	4.48(2.00~10.02)	<0.01	Fixed effects model	3.05(1.93~4.83)	<0.01
Fewer years of working experience	(Niu, 2022)	Random effects model	1.32(1.08~1.62)	<0.01	Random effects model	1.35(1.08~1.68)	<0.01
Fear of COVID-19	(Lai, 2020)	Random effects model	1.81(1.42~2.89)	0.012	Random effects model	1.47(1.03~2.12)	<0.01
High education	(Yin, 2020)	Random effects model	0.58(0.37~0.91)	0.01	Random effects model	0.72(0.53~0.96)	<0.01
Outside Hubei province	(Yang, 2022)	Random effects model	0.54(0.41~0.72)	<0.01	Fixed effects model	0.61(0.50~0.75)	<0.01
Social support	(Xia,2021)	Random effects model	0.44(0.17~1.15)	<0.01	Random effects model	0.55(0.18~1.68)	<0.01
Psychological resilience	(Wang, 2020)	Random effects model	0.97(0.91~1.02)	<0.01	Fixed effects model	0.97(0.96~0.99)	<0.01

### Publication bias

The results of Egger’s test and Begg’s test showed P>0.05 for all the influences except fewer years of working experience, social support and psychological flexibility, indicating that there was no publication bias for the other influences (**Table 5** **).** The results of Egger’s test for fewer years of working experience, social support and psychological flexibility were P=0.000, 0.047 and 0.042, respectively, suggesting the possibility of publication bias. Correcting for publication bias by the cut-and-patch method, it was found that there was no significant change in their combined effect size estimates before and after the cut-and-patch, indicating that publication bias had little effect and their results were relatively stable.

**Table 5:** Publication bias of factors affecting PTSD among Chinese healthcare workers during the COVID-19 pandemic.

Influencing factors	Egger’s test		Begg’s test		
	*t*	*P*	Kendall Score	*Z*	*P*
Female	4.12	0.151	3	1.04	0.296
Married	-1.34	0.409	-3	1.04	0.296
Fewer years of working experience	38.14	0.000	6	1.22	0.221
Fear of COVID-19	3.48	0.074	2	0.34	0.734
High education	-9.53	0.067	-3	1.04	0.296
Outside Hubei province	-6.87	0.092	-3	1.04	0.296
Social support	-13.4	0.047	-1	0.00	1.000
Psychological resilience	-15.24	0.042	-3	1.04	0.296

## Discussion

### Main findings

In this study, we conducted a Meta-analysis of the prevalence and influencing factors of PTSD in Chinese healthcare workers during COVID-19.Because COVID-19 first outbreak in China, and for a long time has been adopted positive strict epidemic prevention policy, Chinese medical staff directly contact with patients, facing greater risk of life safety, more vulnerable to all kinds of epidemic information and more likely to be excessive involved in the outbreak, so in the medical staff produced more obvious post-traumatic psychological stress response. For Chinese health care workers during COVID-19, they are directly exposed to the life-threatening risk caused by this fatal disease. In other words, for healthcare providers during COVID-19, they met the basic requirement for a PTSD diagnosis of experiencing a life-threatening or extremely stressful event. As a result, health care workers suffer from post-traumatic stress disorder. Adaptation disorders and stress-induced syndrome are more common in the general population who are not directly exposed, which does not belong to the group included in this study. Twenty-one studies with high quality literature, involving multiple provinces in China, were representative. The results showed that the total prevalence of PTSD among Chinese medical workers during COVID-19 was 29.2% (95% CI: 20.7% -37.7%), which was higher than cases in the general population (38) and infected patients (39). The prevalence of PTSD among healthcare workers during SARS (40), Middle East Respiratory Syndrome (MERS) (41), and non-epidemic periods was lower than that during the COVID-19 epidemic (42). In a study involving a global population of healthcare workers, the incidence of PTSD among Chinese healthcare workers was higher than that in the UK (43), Turkey (44), and at the same time, was also higher than the overall prevalence of PTSD (45). This indicates that the psychological trauma caused by COVID-19 to health care workers in China is very serious, who are always in the front line of the fight against the epidemic, are in a threat environment for a long time, and are more vulnerable than other groups. This suggests that more attention should be paid to the psychological status of Chinese healthcare workers and provide timely psychological counseling.

### Risk factors

In this study, eight factors were found to be risk factors for PTSD in Chinese healthcare workers during COVID-19. Women are more sensitive and less psychologically resilient in the face of emergencies (46, 47), and have a higher rate of post-traumatic fear, panic, helplessness, body anxiety sensitivity, and dissociation, and are therefore more prone to developing PTSD (48). Nurses are at a higher risk of infection as they are in closer contact with patients and interact for longer periods of time than other healthcare workers. Providing direct care to patients makes them more susceptible to emotions associated with pain and fear of death, which also increases their risk of PTSD (49, 50). Marriage was associated with PTSD among Chinese healthcare workers during COVID-19, which found that married healthcare workers were more concerned about their own health status and the health of their family members, had a heavier burden of caring for their family members, experienced more trauma, and consequently had much more severe PTSD symptoms (32, 35). Frontline healthcare workers are the closest contacts of patients with CKP(Classical Klebsiella pneumoniae), have the highest chance of being infected and are more likely to be in a subhealthy state. Most of them experience psychological stress as a result of their high-intensity work, which makes them more likely to suffer from PTSD (51).

It has been found that fewer years of working experience is associated with PTSD in the face of an epidemic (52, 53). This may be due to the fact that most of the healthcare workers with fewer years of working experience have young children, need to take care of their work and family at the same time. This requires a lot of energy and concerns that their family members will have an increased risk of infection because of the nature of their work (54). In addition, most of them have not participated in public health emergencies such as SARS and H1N1 influenza epidemics, lack relevant experience, may have lower self-efficacy at work, and will face greater occupational pressure in the face of epidemics, which makes them more susceptible to stress reactions (17).

Since the outbreak of the novel coronavirus pneumonia epidemic, the high-risk work environment has greatly stimulated the psychology of healthcare workers, making them fearful of contracting the novel coronavirus and even exacerbating their PTSD (55, 56). In addition, the diagnosis of COVID-19 by a family member or friend may place a heavy emotional burden on healthcare workers (31), and these trends can be explained by peer support for adaptive coping. Chronic illnesses are risk factors for the development of serious diseases, and pre-existing illnesses lead to their impaired overall health and susceptibility to infections. The vicious cycle effect between PTSD and diseases such as COVID-19 can be demonstrated by a two-stage stress response model of PTSD, which can increase susceptibility to infection through chronic stress (57). Therefore, COVID-19 leads to elevated PTSD among Chinese healthcare workers (58).

### Protective factors

In the early 2020s, the novel coronavirus pneumonia epidemic was the first to break out in China, and Hubei province in China was at the center of the epidemic, with a significantly higher number of confirmed and severe cases than other provinces in China. As a result, the workload and work intensity of healthcare workers in Hubei Province were much greater than in other provinces (25), and healthcare workers outside Hubei Province were less likely to develop PTSD.Studies have pointed out that highly educated healthcare workers are more likely to have access to adequate information about COVID-19, have a greater store of clinical knowledge, and have a greater ability to deal with problems and resist stress when faced with situations of high-intensity work pressure (27). Several studies have shown that psychological resilience predicts secondary traumatic stress in healthcare workers and that psychological resilience is influenced by training or experience and has positive effects on psychological outcomes (45). Meanwhile, psychological resilience plays a mediator between COVID-19 stress experience and acute stress disorder (59). Social support is an important protective factor for healthcare workers suffering from PTSD during COVID-19, and it has been shown that the provision of adequate social support may help to reduce the incidence of adverse psychological symptoms such as depression, anxiety and PTSD (60). Adequate coworker support is beneficial in reducing work-related fatigue, and the more support from leaders, the fewer posttraumatic stress reactions (61). One study showed that healthcare workers with social support were 49% less likely to develop PTSD (62). These findings further emphasize the importance of social support for healthcare workers to maintain good mental health.

## Limitations

This study has several limitations. First, the search strategy we used may be flawed, leading in the omission of some studies that met the inclusion criteria. If we could use the asterisk after the radical root of the word to extend the search strategy, as in the protective factor *, health work *, then the number of articles would increase substantially. We suggest that this strategy can be used in future updates to improve the accuracy of article results. Moreover, we only included the public literature, excluded the literature with quality assessment less than and equal to 4 points, and were unable to extract ORs and 95%ci, which may have some influence on the results. Second, the included studies used different assessment tools and there may be some heterogeneity between studies as a result. Nine of the 21 studies included in the analysis in this article used the revised Event Impact Scale (IES-R) as a diagnostic assessment tool).12 articles used the PTSD Urban Resident Version Inventory (PCL-C) or PTSD Inventory DSM-5 (PCL-5) to diagnose PTSD among Chinese health care workers during the COVID-19 pandemic. The Event Impact Scale Revision (Impact of Event Scale-Revised, IES-R) assessed medical staff response after exposure to a traumatic event. The scale has 22 entries, including three dimensions: intrusion, arousal and escape. Using the grade linkert5 level scoring method, 0 to 4 points represent “no effect” to “serious impact” points, respectively, and the total score ranged from 0 to 88 points (63). The scale has good reliability and validity, and the Alpha value of the internal consistency coefficient is from 0.89 to 0.96.In China, many studies have applied IES-R to the PTSD assessment of people after natural disasters and other traumatic events to conduct the reliability and validity test. It has higher reliability compared to other tools, so they prefer to use this scale.Qi et al. (2022) (64) conducted a meta-analysis of the prevalence of PTSD among medical workers in different countries during COVID-19.14 articles using IES-R as a measurement tool, including France, Britain, Italy, China, Korea, the Netherlands, and Ethiopia, evaluated PTSD symptoms during COVID-19. Furthermore, in the meta-analysis of risk factors for PTSD in Brewin et al. (2000) (65), PTSD could be assessed using the IES-R scale. This indicates that the application of the IES-R scale in the field of research PTSD, which is used to assess the subjective distress of a specific traumatic event has a high degree of recognition. In the first time of the COVID-19 outbreak, unknown and life-threatening highly infectious viruses cause dramatic changes in the level of psychological quality of health care workers directly exposed to this risk. It is also noteworthy that some other studies in China did not specify the time frame requiring respondents to score traumatic stress symptoms or, not assessing symptoms experienced in the past month, but also focused on symptoms in the past week, suggesting that some researchers may focus on acute rather than sustained stress. Taken together, both IES-R and PCL-5 can be used to assess PTSD, with only different focus, and two different assessment tools may create bias to define PTSD outcomes, which is also the source of heterogeneity elaborated in the limitations of this paper. The source of heterogeneity in this paper may be the difference in PTSD diagnostic scale, similarly, which also suggests us that in the future public emergency psychological investigation research, we need to improve the research methods and choose screening tools compatible with psychological research for measurement. Third, the influencing factors covered by the studies are not the same, and certain influencing factors such as sleep quality are less studied and cannot be analyzed. Finally, the evaluation indexes of this study may be biased by subjective factors, resulting in certain effects.

## Conclusions

During the novel coronavirus pneumonia epidemic, Chinese healthcare workers have been at the forefront of operations against COVID-19, and their PTSD was influenced by a combination of factors, including frontline work and fear of COVID-19. The results of this study may help healthcare organizations better understand the PTSD of healthcare workers during the new coronavirus pneumonia pandemic and provide appropriate measures.

## Data availability statement

The original contributions presented in the study are included in the article/supplementary material. Further inquiries can be directed to the corresponding author.

## Author contributions

MZ: Data curation, Formal analysis, Software, Writing – original draft. MB: Formal analysis, Data curation, Software, Writing – original draft. HW: Conceptualization, Data curation, Writing – original draft. WF: Project administration, Resources, Supervision, Writing – review & editing. LK: Project administration, Resources, Supervision, Writing – review & editing. CZ: Software, Supervision, Writing – review & editing. ZZ: Project administration, Resources, Supervision, Writing – review & editing.
